# Transvenous right ventricular pacing in a patient with tricuspid mechanical prosthesis

**DOI:** 10.1186/1749-8090-3-42

**Published:** 2008-07-09

**Authors:** Juan Sierra, José Rubio

**Affiliations:** 1 Service of Cardiovascular Surgery, University hospital, Santiago de Compostela, Spain; 2 Framan – Bugallido, 15866, La Coruña, Spain

## Abstract

We report a patient in whom permanent endocardial pacing was accomplished by passage of the electrode through a mechanical tricuspid valve. Echocardiography study showed a minimal tricuspid regurgitation.

## Case report

A 66 year-old woman was admitted to our hospital, because she had an episode of cardiovascular collapse requiring cardiopulmonary resucitation four days after cardiac operation in other hospital. The patient had previously undergone one closed and three open heart procedures. The first operation was a closed mitral surgery by a left thoracotomy. After nine years she needed new surgery because she had mitral stenosis and a mitral comisurotomy was done throught a sternotomy and under cardiopulmonary bypass. Ten years after the second operation, because she had mitral stenosis with extensive calcification of the anterior leaflet and aortic and tricuspid regurgitation, a double mitro-aortic valve replacement was done with two Bjork- Shiley (Shiley Inc – Irvine. CA, USA) prostheses. Also a De Vega tricuspid annuloplasty was performed. In 2002 symptoms of congestive heart failure, dyspnea and severe tricuspid insufficiency necessitated replacement of the tricuspid valve by a CarboMedics Prosthesis (CarboMedics Inc, Austin, Tex – USA). This operation was performed through right thoracotomy. The patient was discharged from the hospital one week later.

Four days later, she was admitted to our hospital because she had an episode of cardiovascular collapse requiring cardiopulmonary resucitation. The physical examination was remarkable for elevated jugular venous pressure.

Chest radiography revealed the presence of important bilateral pleural effusion and a right ventricle enlargement. The ECG showed atrial fibrillation with a ventricular rate of 35 beats/min. No Digoxin or Beta Blocker treatment was present.

Transthoracic echocardiography demostrated a normal function of the three prosthesis and a decreased right ventricular function.

**Figure 1:**
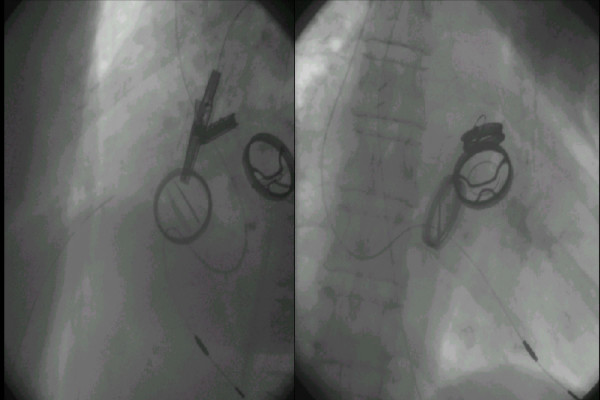
Two different projections with the lead through the tricuspid prosthesis. Only one leaflet is affected.

The patient who was on an anticoagulant therapy with coumadin, was put on a regimen of heparin. After three days and to avoid a new thoracotomy(Fifth) a left ventricular pacing with an endocardial lead through the coronary sinus was performed. The stimulation threshold there was 4 V at 0,5 ms and the R wave amplitude was 10 mV. New positions were tried and due to the high stimulation threshold, we decided to place the lead in the right ventricle through the tricuspid prosthesis (fig 1). The stimulation Threshold was 0,6 V at 0,5 ms. The R wave amplitude was 16 mV, the impedance was 700 om. The electrocardiogram showed normal ventricular pacing, the lead was connected to a single chamber rate adaptative pacemaker (Kairos SR. Biotronik) which was placed in a subfascial prepectoral pocket. After a week an x-ray revealed that the lead position remained stable.

Transesophageal echocardiography showed a mild tricuspid regurgitation with a pulmonary artery pressure of 60 mmHg and a right ventricle with a better function than before the implantation.

## Discussion

In this article we reported the first case, to our knowledge, of a transvenous right ventricular pacing in a patient with tricuspid mechanical prosthesis and multiple previous heart surgeries. Patients undergoing tricuspid valve replacement frequently require permanent pacing and if a permanent pacemaker was not implanted at the time of valve replacement, a new thoracotomy would be necessary for epicardial lead implantation. A transvenous lead implantation with Ventricular pacing from the coronary sinus is a feasible approach in patients with tricuspid mechanical prosthesis [1], but as was described by Lee [2] acute stimulation thresholds are higher and this was the case with our patient.

The endoscopic surgery is another possibility, but patients with obvious dense adhesions not be scheduled for an endoscopic procedure [3] because a failure during surgery requires obligatory conversion to an open procedure and may be exceptionally dangerous in patients with multiple previous cardiothoracic surgeries.

Pernenkil [4] described a permanent endocardial pacing through a porcine bioprosthetic tricuspid valve without problems in a long term follow-up, but as was described by Winter [1], transvenous right ventricular endocardial pacing is contraindicated in patients with tricuspid mechanical prosthesis and this was true with the use of old generation prostheses (monodisc).

With bileaflet prosthesis it is possible to pace the right ventricle without important hemodynamics alterations. Since only one leaflet is affected, as in our case, the tricuspid regurgitation is minimal.

## Authors' contributions

JS and JR were the surgeons. All authors read and approved the final manuscript.

## Consent

Written informed consent was obtained from the patient for publication of this case Report and accompanying images.
